# Getting Real: How Intersectionality Challenges the ‘One Size Fits All’ Approach to Global Mental Health

**DOI:** 10.1111/jpm.70031

**Published:** 2025-09-18

**Authors:** Zaqia Rehman, Paul Illingworth

**Affiliations:** ^1^ Leicester School of Allied Health Sciences, Faculty of Health and Life Sciences De Montfort University Leicester UK

**Keywords:** climate change, inclusivity, intersectionality, mental health

## Abstract

This paper offers an intersectional perspective to enable inclusivity when addressing global mental health challenges, whilst also tackling social exclusion and vulnerability. Examples are given from purposefully selected countries to demonstrate the effectiveness of exploring issues through an intersectional approach. The authors conclude that a ‘one size fits all’ approach, when working towards universal mental health, is not the best approach and cannot provide a solution for all. They suggest the WHO, UN, governments and mental health agencies need to view global mental health differently to achieve alternative and better targeted solutions.

## 1 Introduction

Globally, governments, cities, towns, villages, communities and individuals are exploring ways to adapt to climate change and its impact on mental health. It is widely acknowledged there are multiple factors which impact on mental health; these are compounded by the crises in care, gender and racial abuse and discrimination, socio‐economic inequality and recently the global COVID‐19 pandemic. Addressing these factors individually reduces the effectiveness of working towards resilience and the vision of addressing the global mental health crisis. In addition, this also requires the correct data, strategies and implementation to reduce risk and build adaptation and resilience to what is already a deeply divided economic, social and political entity. Consequently, it is increasingly important that key organisations such as the World Health Organisation (WHO), United Nations (UN), Governments, policymakers, planners and professionals utilise intersectional frameworks which confront dominant models and inequalities and transform holistically sustainable universal global mental health.

This paper offers an intersectional perspective to enable an inclusive approach when addressing global mental health challenges at the backdrop of social exclusion and vulnerability. The authors argue that using the term ‘global’ does not necessarily equate applicability to low‐ and middle‐income countries (LMICs) and draw on the overlapping social categories to illustrate barriers in a ‘one size fits all’ approach to mental health.

Originating in the 1980s, (Crenshaw 1988) intersectionality draws from critical race theory and feminist studies to address how overlapping and multiple dimensions of social identities form systems of oppression or privilege. For example, intersectionality acknowledges how the intersections of social characteristics such as gender, race, class, age, disability and sexual orientation shape the experiences of privilege, discrimination and oppression (Ahmed 2017; K. Crenshaw 1989). A single view on the multifaceted nature of global mental health, such as a medical or psychological model, for example, gives little room to address complex issues (Collins and Bilge 2020). Issues in global mental health can be caused or aggravated by climate change that will pattern differing outcomes through intersections of gender, race, location and sexuality. Therefore, one universal model of care is not likely to result in positive outcomes globally.

### 1.1 Intersectional Perspectives

Intersectionality is both a framework and practice and has generated a great deal of momentum in both law and social sciences, especially in the last 25 years. The concept of intersectionality was initially focused on women of colour in the United States of America. However, its use by Black feminists especially in the United States extended its use into identity and marginalised groups (Combahee River Collective 1978). K. Crenshaw's (1991) work around ‘mapping the margins of intersectionality’, was probably the point at which intersectionality took a more central stage in social political movements and there was a greater focus on categories of difference around racialisation, dominance, sexuality and marginalisation. For example, K. Crenshaw (1989) argued that Critical Race Theory focussed on Black men and overlooked patriarchy, while feminist scholarship theorised the experiences of White women and illustrated how the liminal occupation of single axis frameworks of antidiscrimination law (e.g., racism or sexism) produced the subordination of Black women in both spheres. The framework highlights how identities (e.g., race, gender, class, Indigenous, disability) are mutually constitutive and recognises the interlocking nature of the social categories (e.g., black/white, man/women, middle/low class) that create a unique situation of marginalisation and privilege (Collins 1999; Shields 2008; Veenstra 2011). Thus, intersectionality defines identities as analytically inseparable (Veenstra 2011) and seeks to understand complex experiences by challenging socially and politically informed categories (Hancock 2007).

Previous research (Heinz and Brandt 2024) show that social and economic inequalities are exacerbated due to the indirect impact of climate change (e.g., food insecurity, housing, displacement, lack of access to clean air and water pollution). Yet, these ‘exacerbations’ at the intersection of disability × gender (e.g., man non disability/women with disability) pattern different mental health experiences, explicating new dimensions of marginalisation and privilege (Collins 1999) rooted in patriarchal and political systems. The paper draws on the intersectional perspective to consider the applicability and effectiveness of a universal mental health action plan and reflects on current contextual issues by reflecting on six countries.

### 1.2 Global Mental Health in an Intersectional Context

The United Nations developed the Sustainable Development Goals (SDGs) as a ‘blueprint’ for global action, to generate a more equitable and sustainable world. Mental health is a significant aspect of these, as the World Health Organization (WHO 2019) acknowledged, ‘there can be no health or sustainable development without mental health’. Yet despite the WHO and other organisations such as the UN and governments globally making some progress and the WHO Mental Health Action Plan (MHAP) having been first established in 2013 (WHO 2013) later extended to 2030 (WHO 2021), there is clearly much more work needed. The WHO Mental Health Atlas (WHO 2020) is a ‘normative tool, originally published in 2001 and has been regularly updated. The Atlas monitors WHO's member states progress on objectives, and since the establishment of the MHAP, those targets also. The WHO website shows data collected from 171 out of the overall member states.

The authors compared six countries, five in what are known as a LMICs and one classed as a higher income country (HIC);

**Table:** 

Low income	Sierra Leone
Lower middle income	Pakistan, Philippines and Sri Lanka
Upper middle country	Malaysia
High‐income country	United Kingdom

Before comparing the data, it is important to state that the authors acknowledge these groupings are, what Collins (1986) called ‘oppositional thinking’ in which people are conceptualised within an inescapable hierarchy; in other words one is perceived as superior, the other(s) inferior. High income being perceived as the norm, in policies and academic writing. It is fascinating to note that in the WHO Mental Health Atlas, there are further sub‐divisions into upper and lower middle income. But are high‐income countries always best? High‐income countries are responsible for much of the climate issues and impact on low‐income countries, reinforcing the power play.

Further exploration of some of the data sets against these countries is illuminating. In the Philippines, the ratio of mental health workers per 100,000 population rose slightly from 1.15 in 2014 to 1.68 in 2020 (although it was 1.70 in 2017). Sri Lanka also showed growth, from 3.44 in 2014 to 5.46 in 2020. Whereas in Malaysia, the figures are 2.64 in 2014, rising to 8.92 in 2017, then falling to 5.86 in 2020. Yet in Pakistan, it significantly reversed from 19.15 in 2014 to 0.55 in 2020. It is difficult to say why that might be, and there have likely been many factors influencing it, but the fragility of the political state of the nation may have been a major contributing factor. Sierra Leone also had a significant drop, and of those highlighted, it has the lowest ratio, 1.34 in 2014, dropping to 0.34 in 2020. In comparison, the United Kingdom, the ratio was 106.10 per 100,000 in 2014 and 201.14 in 2020. This may reflect the numbers of people with a diagnosed mental illness or the dominant model of delivery, but it also exemplifies inequity in accessing resources.

Alternatively, this may reflect a more Northern white medical treatment model being the key driver of service development and delivery. It might even be questioned whether the metrics used by the WHO for the Mental Health Atlas are themselves based on the needs of majority white populations and may not address critical issues such as different experiences of those with mental health issues in countries that criminalise suicide (there are still 20 countries who criminalise suicide globally), or there are more widely accepted views of stigmatisation and abuse of people with mental illness. Yet, universal MHAP indicators, which currently operate in a Eurocentric framework, rely on existing infrastructure to shoehorn mental health and promotion initiatives in LMIC's. Mental health research shows the importance of community and familial context in positive mental health outcomes (Bosqui et al. 2024). Yet, the indicators are not centred around the collectivist cultural context.

Health promotion in LMICs is consistently underreported within mainstream research, leading to the homogenisation of Eurocentric policies serving LMICs. Within the Global South discourse, social disadvantage and community connectedness play a critical role in health outcomes, particularly in the context of community engagement when designing and delivering health services (Epling et al. 2025). Importantly, community‐based interventions have improved outcomes for cardiovascular diseases (Hassen et al. 2021), reduced neonatal deaths (Lassi et al. 2015) and improved mental health service engagement (Giebel et al. 2024). These examples illustrate the importance of context‐specific interventions for better health outcomes.

This paper does not undermine the importance of the MHAP targets but calls for greater reflection on the context‐specific norms, values and representations associated with mental illness which shape the experiences and likelihood of accessing mental healthcare. For example, most of the LMICs discussed in this paper align with the collectivist cultural traits, where caregiving is seen as a familial obligation (Scharlach et al. 2006). Thus, increasing the inpatient mental health facilities as per MHAP might not be appropriate for these communities. Nonetheless, the lack of resources and infrastructure in mental health care services hinders implementation and prioritisation of meeting the targets, as evidenced in Table 1. Albeit one needs to question, and perhaps this is where a critical theory framework can benefit, whether the UK mental health services are driven by privilege (e.g., privilege to prioritise mental health).

**TABLE 1:** Comparison of the seven countries mental health promotion & prevention programmes.

Mental health promotion and prevention programmes	Malaysia	Pakistan	Philippines	Sierra Leone	Sri Lanka	United Kingdom
Suicide prevention programme	—	—	Yes	—	Yes	Yes
Mental Health Awareness/Anti‐stigma	No	—	—	Yes	Yes	Yes
Early Child Development	—	—	—	—	Yes	Yes
School‐based mental health prevention and promotion	Yes	Yes	—	—	Yes	Yes
Parental/Maternal mental health promotion and prevention	—	—	—	—	Yes	Yes
Work‐related mental health prevention and promotion	Yes	—	—	Yes	Yes	Yes
Mental health and psychosocial component of disaster preparedness, disaster risk reduction	Yes	Yes	Yes	Yes	Yes	—

Again, the same six countries have been selected to compare, but the indicators demonstrate the stark difference across countries. For example, it is worth noting that Malaysia, Pakistan, the Philippines, Sierra Leone and Sri Lanka all had mental health promotion, prevention, disaster preparedness and risk reduction in place. The objective is prioritised in these contexts, considering that these countries have been exposed to natural disasters, whereas the United Kingdom has not. It will be interesting to see at the next release of data (post COVID‐19) whether the United Kingdom has instigated one. What is encouraging is that Sri Lanka was the only country, of the six purposively selected, to rate as providing mental health promotion and prevention in all seven areas.

The illustrative comparison of the mental health promotion and prevention programmes depicts superficiality and omits explicit actions to respond to the unique social climate change issues in LMIC, namely poverty. More detail is provided in the WHO MH Atlas. LMIC countries dependent on agriculture, farming or livestock are excluded from ‘Work‐related mental health prevention and promotion’, which is primarily monitored and implemented in corporate organisations through governmental incentivisation. For example, people living in rural areas in Pakistan, the Philippines and Sierra Leone or those who rely on agriculture and livestock are compounded with multiple issues at the intersection of poverty and climate change, increasing susceptibility to poor mental health outcomes within a private working climate. In the west, this would be deemed as self‐employment. Mental health care in Sierra Leone is scarce despite thousands suffering post‐traumatic stress following the war and the Ebola epidemic; there is high drug and alcohol abuse as a result (Amnesty International 2021). With regards to Sierra Leone, the authors acknowledge it might be different, as they are big producers of rice and that is a significant employer of people. But they also rely on the mining of diamonds and are rich in other natural resources, including gold and bauxite, which is made into titanium ore, plus aluminium ore.

## 2 Conclusion

This paper offers a brief, purposeful intersectional exploration of global mental health. It by no means offers a deep dive into all the various intersections impacting people's/communities' mental health. It does show, however, that a ‘one size fits all’ approach cannot do justice to the multifactorial causation of mental ill‐health, and will not take into account any cultural and local nuances, which are core to, not only the cause, but also the solution. The paper also demonstrates that viewing mental health through a dominant western model will not give an accurate picture of causation to allow an appropriately designed preventive and intervention service that would lead to universal mental health for all.

The WHO, UN, governments and mental health agencies should reflect on their current approaches/practices. They must consider whether what they are currently suggesting and/or implementing is truly helping those with mental health issues globally. And if not, explore what they are doing through an intersectional perspective, which will give alternative and better targeted solutions.

## Ethics Statement

The authors have nothing to report.

## Conflicts of Interest

The authors declare no conflicts of interest.

## Data Availability

Data sharing not applicable to this article as no datasets were generated or analysed during the current study.

## References

[jpm70031-bib-0001] Ahmed, S. 2017. Living a Feminist Life. Duke University Press.

[jpm70031-bib-0002] Amnesty International . 2021. “They Are Forgetting About Us.” The Long‐Term Mental Health Impact of War and Ebola in Sierra Leone. Index Number: AFR 51/4095/2021. https://www.amnesty.org/en/documents/afr51/4095/2021/en/.

[jpm70031-bib-0003] Bosqui, T. , A. Mayya , S. Farah , et al. 2024. “Parenting and Family Interventions in Lower and Middle‐Income Countries for Child and Adolescent Mental Health.” Comprehensive Psychiatry 132: 152483. 10.1016/j.comppsych.2024.152483. 38631272

[jpm70031-bib-0005] Collins, P. H. 1986. “Learning From the Outsider Within: The Sociological Significance of Black Feminist Thought.” Social Problems 33, no. 6: s14–s32. 10.2307/800672.

[jpm70031-bib-0023] Collins, P. H. 1999. “Reflections on the Outsider Within.” Journal of Career Development 26, no. 1: 85–88. 10.1177/089484539902600107.

[jpm70031-bib-0006] Collins, P. H. , and S. Bilge . 2020. Intersectionality. John Wiley & Sons.

[jpm70031-bib-0022] Crenshaw, K. W. 1988. “Race, Reform, and Retrenchment: Transformation and Legitimation in Antidiscrimination Law.” Harvard Law Review 101, no. 7: 1331. 10.2307/1341398.

[jpm70031-bib-0007] Crenshaw, K. 1989. “Demarginalizing the Intersection of Race and Sex: A Black Feminist Critique of Antidiscrimination Doctrine, Feminist Theory and Antiracist Politics.” In Feminist Legal Theories, edited by K. Maschke , 1st ed. Routledge. 10.4324/9781315051536.

[jpm70031-bib-0008] Crenshaw, K. 1991. “Mapping the Margins: Intersectionality, Identity Politics, and Violence Against Women of Colour.” Stanford Law Review 43, no. 6: 1241–1299. https://blogs.law.columbia.edu/critique1313/files/2020/02/1229039.pdf.

[jpm70031-bib-0009] Epling, S. W. , A. M. Bjork , L. M. Cruz , and M. C. Baker . 2025. “Approaches to Increase Access to Community‐Based Infectious Disease Control for Ethnically, Racially, and Religiously Marginalised Populations: A Scoping Review.” Lancet Infectious Diseases 25, no. 5: e269–e279. 10.1016/S1473-3099(24)00744-8. 39922209

[jpm70031-bib-0010] Giebel, C. , M. Gabbay , N. Shrestha , et al. 2024. “Community‐Based Mental Health Interventions in Low‐and Middle‐Income Countries: A qualitative Study With International Experts.” International Journal for Equity in Health 23, no. 1: 19. 10.1186/s12939-024-02106-6. 38308294 PMC10835969

[jpm70031-bib-0011] Hancock, A. M. 2007. “When Multiplication Doesn't Equal Quick Addition: Examining Intersectionality as a Research Paradigm.” Perspectives on Politics 5, no. 1: 63–79.

[jpm70031-bib-0012] Hassen, H. Y. , R. Ndejjo , G. Musinguzi , J. P. Van Geertruyden , S. Abrams , and H. Bastiaens . 2021. “Effectiveness of Community‐Based Cardiovascular Disease Prevention Interventions to Improve Physical Activity: A Systematic Review and Meta‐Regression.” Preventive Medicine 153, no. 106: 797. 10.1016/j.ypmed.2021.106797. 34508731

[jpm70031-bib-0013] Heinz, A. , and L. Brandt . 2024. “Climate Change and Mental Health: Direct, Indirect, and Intersectional Effects.” In The Lancet Regional Health–Europe. WHO (2013) Mental Health Action Plan 2013–2020. WHO. 10.1016/j.lanepe.2024.100969 PMC11360162 39210948

[jpm70031-bib-0014] Lassi, Z. S. , P. F. Middleton , C. Crowther , and Z. A. Bhutta . 2015. “Interventions to Improve Neonatal Health and Later Survival.” An Overview of Systematic Reviews. eBio Medicine 2, no. 8: 985–1000. 10.1016/j.ebiom.2015.05.023. PMC4563123 26425706

[jpm70031-bib-0015] Scharlach, A. E. , R. Kellam , N. Ong , A. Baskin , C. Goldstein , and P. J. Fox . 2006. “Cultural Attitudes and Caregiver Service Use: Lessons From Focus Groups With Racially and Ethnically Diverse Family Caregivers.” Journal of Gerontological Social Work 47, no. 1–2: 133–156. 10.1300/J083v47n01_09. 16901881

[jpm70031-bib-0016] Shields, S. A. 2008. “Gender: An Intersectionality Perspective.” Sex Roles 59, no. 5–6: 301–311.

[jpm70031-bib-0017] The Combahee River Collective . 1978. “The Combahee River Collective Statement.” Zillah Eisenstein. Cited in BlackPast, B. (2012). (1977) The Combahee River Collective Statement. BlackPast.org. https://www.blackpast.org/african‐american‐history/combahee‐river‐collective‐statement‐1977/.

[jpm70031-bib-0018] Veenstra, G. 2011. “Race, Gender, Class, and Sexual Orientation: Intersecting Axes of Inequality and Self‐Rated Health in Canada.” International Journal for Equity in Health 10, no. 1: 3. 21241506 10.1186/1475-9276-10-3 PMC3032690

[jpm70031-bib-0019] WHO . 2019. The WHO Special Initiative for Mental Health (2019–2023): Universal Health Coverage for Mental Health. WHO. https://iris.who.int/bitstream/handle/10665/310981/WHO‐MSD‐19.1‐eng.pdf?sequence=1&isAllowed=y.

[jpm70031-bib-0020] WHO . 2020. Mental Health World Atlas 2020. WHO. https://www.who.int/publications/i/item/9789240036703.

[jpm70031-bib-0021] WHO . 2021. Comprehensive Mental Health Action Plan 2013–2030. WHO.

[jpm70031-bib-0024] World Health Organization . 2013. Mental Health Action Plan 2013–2020. World Health Organization. https://www.who.int/publications/i/item/9789241506021.

